# Active Vibration Control of a Piezo-Bonded Laminated Composite in the Presence of Sensor Partial Debonding and Structural Delaminations

**DOI:** 10.3390/s19030540

**Published:** 2019-01-28

**Authors:** Asif Khan, Heung Soo Kim

**Affiliations:** Department of Mechanical, Robotics and Energy Engineering, Dongguk University-Seoul, 30 Pil-dong 1 Gil, Jung-gu, Seoul 04620, Korea; khanuet11@gmail.com

**Keywords:** piezo-bonded laminated composite, active vibration control, sensor partial debonding, structural delamination

## Abstract

In this paper, the active vibration control of a piezo-bonded laminated composite is investigated in the presence of sensor partial debonding and structural delamination. Improved layerwise theory, higher-order electric potential field, and the finite-element method were employed to develop an electromechanically coupled model for the two types of damage (i.e., sensor partial debonding and delamination). The developed model was numerically implemented on a single-input-multi-output (SIMO) system to demonstrate the effects of sensor partial debonding and structural delamination on the ability of a constant gain velocity feedback (CGVF) controller to attenuate vibration. The two types of damage were assessed in terms of controlled outputs of the sensors, nodal displacements, and control input signals being applied to the actuator to suppress vibrations. The obtained results showed that the sensor partial debonding and structural delamination have opposite effects on the vibration-attenuation characteristics of the CGVF controller. The presence of partial debonding in the sensor made the controller less able to suppress vibrations because of a spurious sensing signal, whereas structural delamination increased the control authority of the controller because of the loss of structural stiffness that results from structural delamination.

## 1. Introduction

Smart composite laminates are obtained by integrating smart materials (PZTs, shape memory alloys, fiber optics) with laminated composites in the form of surface bonded or embedded sensors and actuators. Smart composite laminates synergistically integrate the preferential properties of the host laminates (e.g., high specific strength, high specific stiffness, fatigue resistance, design flexibility) and the smart sensing and actuation capabilities of the smart materials. Some contemporary engineering applications of the smart composite laminates are in the aerospace, automotive, marine, and robotics fields [1,2,3], among others Although smart composite laminates offer the advantages of self-sensing [4], diagnostics [5], vibration and noise suppression [6,7], shape control [8], and can respond to changing environments [9], they are also susceptible to various types of damage in the orthotropic host laminates as well as partial debonding failure of the smart elements [10]. Some frequent damages in laminated composites are delamination [11,12], matrix crack [13,14], fiber breakage [15], among others. Delamination is one of the most feared and major modes of failure, because of its serious implications for the integrity of the structure [16]. Also, the high free-edge stress between the host laminate and smart elements ultimately results in the partial debonding failure of the sensor/actuator from the host structure [17].

In the literature, various numerical approaches have been proposed to model the electromechanically coupled problem of a laminated composite with smart elements in the form of sensors and actuators. Mitchell and Reddy [18] proposed a hybrid plate theory for the smart composite laminates. The mechanical displacement field of the host laminate and the electric potential function of the piezoelectric laminae were modeled via a third-order shear deformation theory and a layerwise discretization in the thickness direction, respectively. Crawley and Luis [19] developed static and dynamic analytical models for laminated composites with surface bonded/embedded segmented piezoelectric actuators. Ha et al. [20] presented an FEM-based modeling for the static and dynamic response of piezo-bonded laminated composites subjected to mechanical and electrical excitations. They introduced three-dimensional incompatible modes to account for the global bending behavior caused by the local deformations of piezoceramics. Gohari et al. [21] developed a two-dimensional quadratic multi-layer shell element based on first-order shear deformation theory for the linear strain-displacement static deformation of a laminated composite with macrofiber composite [MFC] actuators. Chattopadhyay and Seeley [22] presented a higher-order theory for modeling composite laminates with induced strain actuators. Alibeigloo and Madoliat [23] incorporated a differential quadrature method and Fourier series approach for the three-dimensional solution of the static analysis of a piezo-bonded cross-ply laminated plate. Li and Shen [24] presented a layerwise finite-element formulation for laminated composite cylindrical shells with piezoelectric layers. Peng et al. [25] proposed a quantitative method for the identification of delamination in carbon fiber/epoxy laminated composite beams. A two-dimensional spectral element method was used to characterize the propagation of lamb waves in an 8-ply composite beam and the fundamental antisymmetric (A_0_) mode was found to be more suitable for damage identification than the fundamental symmetric (S_0_) mode.

The active vibration control of smart composite laminates has turned out to be an indispensable task, because the light weight and flexibility of laminated composite may result in structural instability that could allow undesirably large amplitude vibrations in the structure. Many strategies have been devised for the active control and vibration attenuation of the smart structures, such as direct velocity feedback control [26], LQR control [27], and positive position feedback control [28]. Proulx and Cheng [29] studied the active vibration control of a rectangular plate by using piezoelectric patches of arbitrary shapes and observed that the off-resonance regions were more affected by the shapes of actuators than were the near-resonance regions. Kapuria et al. [30] presented a theoretical framework for modeling active vibration control of piezo-bonded laminated plates by considering the nonlinear behavior of PTZs under a strong electric field. The control input was employed to transform the nonlinear system into an equivalent linear system using feedback linearization. Bendine et al. [31] presented an LQR control algorithm for the active vibration control of a composite plate using discrete piezoelectric sensors and actuators. Zoric et al. [32] studied the fuzzy optimization strategy based on the particle-swarm optimization algorithm for the optimal vibration control of a thin-walled composite beam. Phung-Van et al. [33] investigated the static, free vibration, and dynamic control of smart composite laminates by employing isogeometric analysis and higher-order shear deformation theory. The static deflection and dynamic response of the plates were controlled via displacement and velocity feedback control algorithms, respectively.

Most of the published literature has focused on the active vibration control of the smart composite laminates with perfectly bonded sensors/actuators and pristine host structure. In actual practice, either the smart element may partially debond from the host laminate because of high free-edge stress at the bonding interface [17], or the host laminate may suffer from various types of damage, such as delamination, because of low velocity impacts or manufacturing defects [34,35]. Defects in the host laminate or smart elements may result in an undesirable performance of the controller, or even in an unstable closed loop system. Li et al. [36] proposed a framework based on decoupled error function and the inverse input-output model to detect sensor failures. Raja et al. adapted a sublaminate approach to develop a theoretical model for the static and dynamic analysis of smart composite laminates with damages in the composite substrate and piezoelectric layers. They found that the presence of partial debonding significantly degraded the sensing and actuation capabilities of the smart elements. Kumar et al. [37] and Huang et al. [38] independently studied the effects of partially debonded piezoelectric actuators on the active vibration control of smart composite laminates. Both studies revealed that a partially debonded actuator cannot effectively transfer the electro-elastically generated strain to the host laminate and reduces the ability of the controller to suppress vibration. In another study, partial debonding between the piezoelectric sensor and host laminate was found to reduce the vibration suppression efficiency of the controller [7,39].

This literature review has revealed a gap in research on the relative effects of structural delamination and sensor partial debonding on the active vibration control of the smart composite laminates. We intend to fill this research gap by means of numerical simulations that may help in assessing the critical aspects of structural delamination and partial debonding failure of the smart elements on the active vibration control of smart composite laminates, hoping that our results may help in understanding whether structural delamination or sensor partial debonding is more critical for an active vibration control.

## 2. Theoretical Formulation

The mathematical formulation of a laminated composite with surface bonded or embedded patches of active materials involves a mechanical displacement field, an electric potential field, and electromechanical coupling between the two fields. In this work, we adapted a layerwise displacement field proposed by Kim et al. [40] for the mechanical displacement field, and used a higher-order electric potential field [41] for the potential field of the piezoelectric patches. The electromechanically coupled governing equation was developed via a finite-element method and extended Hamilton’s principle. 

**Figure 1:**
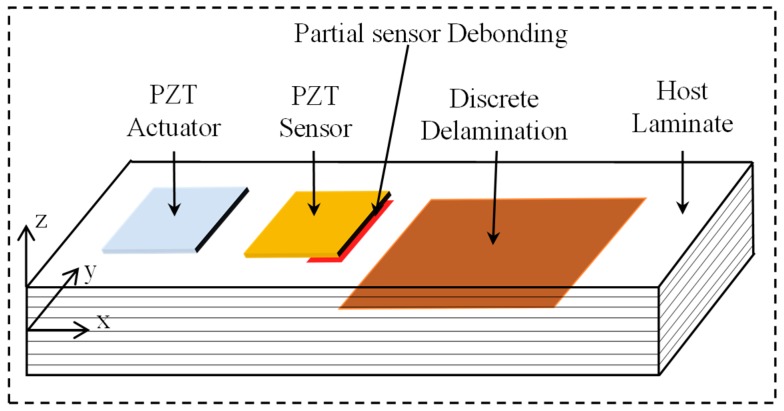
Schematic of a smart composite laminate with partially debonded sensor and discrete delamination.

Figure 1 shows the schematic of a smart composite laminate with two types of defects: partial debonding of sensor, delamination in the host structure. The layerwise displacement field of the problem is shown by Equation (1):

(1) $$\begin{array}{l} U_{y}^{k}=v_{0}+A_{2}^{k}\psi_{x}+B_{2}^{k}\psi_{y}+C_{2}^{k}w_{0,x}+D_{2}^{k}w_{0,y}+E_{2}^{k}\left\{{\bar{w}}_{,x}^{j}\right\}+F_{2}^{k}\left\{{\bar{w}}_{,y}^{j}\right\}+\sum_{j=1}^{N-1}{\bar{u}}_{y}^{j}H(z-z_{j})\\ U_{x}^{k}=u_{0}+A_{1}^{k}\psi_{x}+B_{x}^{k}\psi_{y}+C_{1}^{k}w_{0,x}+D_{1}^{k}w_{0,y}+E_{1}^{k}\left\{{\bar{w}}_{,x}^{j}\right\}+F_{1}^{k}\left\{{\bar{w}}_{,y}^{j}\right\}+\sum_{j=1}^{N-1}{\bar{u}}_{x}^{j}H(z-z_{j})\\ U_{z}^{k}(x,y,z,t)=w_{0}(x,y,t)+\sum_{j=1}^{N-1}{\bar{w}}^{j}(x,y,t)H(z-z_{j}) \end{array}$$

where $U_{x}^{k},\;U_{y}^{k}$, and $U_{z}^{k}$ denote the displacement of a point (*x, y, z*) on the of *k*-th layer along the, *X*, *Y* and *Z* axes of the laminate, respectively. The terms *u*_0_, *v*_0_ and *w*_0_ refer to the displacements of the mid-plane. The quantities of $\psi_{x}$ and $\psi_{y}$ denote the rotations of the normal-to-reference plane about the *x* and *y* axes, respectively and account for the shear deformation along the thickness of the laminate. The terms ${\bar{u}}_{x}^{j},\;{\bar{u}}_{y}^{j}$, and ${\bar{w}}^{j}$ represent the discontinuity in the displacement that is due to in-plane slipping and an out-of-plane separation in the delaminated/debonded regions. *H*(*z − z_j_*) is a Heaviside-unit step function with *z_j_* referring to the delaminated interface. The layerwise coefficients of $A_{i}^{k},\;B_{i}^{k},\;C_{i}^{k},\;D_{i}^{k},\;E_{i}^{k}$ and $F_{i}^{k}$ (*i* = 1, 2) are obtained from the geometric and material properties of the laminate, detail can be found in the reference [40].

The potential variation through the thickness of the piezoelectric patches (working in d_31_ mode) is modelled by higher-order electric potential field as shown by Equation (2):

(2) $$\begin{array}{l} \phi^{p}(x,y,z,t)=\phi_{0}^{p}(x,y,t)-(z-z_{0}^{p})E_{z}^{p}(x,y,t)\;+4{\left(\frac{z-z_{0}^{p}}{h^{p}}\right)}^{2}\\ \;\times \left[\left(z-z_{0}^{p})\left(\frac{{\bar{\phi }}^{p}(x,y,t)}{h^{p}}+E_{z}^{p}(x,y,t)\right)-\phi_{0}^{p}(x,y,t\right)\right] \end{array}$$

where the terms $\phi_{0}^{p}$ and $E_{z}^{p}$ denote the electric potential and the electric field at the mid-plane of the *p*th piezoelectric layer, respectively. The quantity $-(z-z_{0}^{p})E_{z}^{p}$ is used to manage the linear electric-potential distribution through the thickness. The higher-order term refers to the non-uniform potential variation through the thickness, while it also satisfies the equipotential surface-boundary conditions that are prescribed at the electrodes. The term $\phi^{-p}$ refers to the potential difference between the electrodes at the top and bottom of the *p-*th piezoelectric transducer, while $z_{0}^{p}$ and $h^{p}$ denote the position and the thickness of the mid-plane of the *p-*th piezoelectric layer, respectively.

The two fields of Equations (1) and (2) were implemented via finite element method and variational principle was used to obtain the governing dynamic model of the smart composite laminate. Equation (3) shows the finite-element-based electromechanically coupled elemental differential equation of the problem:

(3) $$\left[\begin{array}{cc} M_{u} & 0\\ 0 & 0 \end{array}\right]\left\{\begin{array}{c} {\ddot{d}}_{u}\\ 0 \end{array}\right\}\;+\;\left[\begin{array}{cc} C_{u} & 0\\ 0 & 0 \end{array}\right]\left\{\begin{array}{c} {\dot{d}}_{u}\\ 0 \end{array}\right\}\;+\;\left[\begin{array}{cc} K_{u} & K_{u\varphi }\\ K_{\varphi u} & K_{\varphi } \end{array}\right]\left\{\begin{array}{c} d_{u}\\ d_{\varphi } \end{array}\right\}\;=\;\left\{\begin{array}{c} F_{u}\\ F_{\varphi } \end{array}\right\}$$

where, *M_u_*, *C_u_* and *K_u_* represent the elemental mass, damping and stiffness matrices, respectively. The matrices of *K_uφ_* and *K_φu_* accounts for the electromechanical coupling between the displacement field and electric potential filed. The vectors *d_u_* and *d_φ_* refer to the displacement and electrical unknowns, respectively. The terms *F_u_* and *F_φ_*, respectively denote the external mechanical force vector and electrical input vector.

The governing differential equation was modified into Equation (4) via matrix condensation:

(4a) $$M_{u}{\ddot{d}}_{u}+C_{u}{\dot{d}}_{u}+Kd_{u}=F$$

(4b) $$K=K_{u}-K_{u\varphi }K_{\varphi \varphi }^{-1}K_{\varphi u},\;F=F_{u}-K_{u\varphi }K_{\varphi \varphi }^{-1}F_{\varphi }$$

The presence of delamination in the host laminate is reflected by changes in the stiffness (*K_u_*) and damping (*C_u_*) matrices, whereas the partial debonding of the piezoelectric patches is mainly reflected by variations in the electromechanically coupled stiffness matrices (*K_uφ_, K_φu_*). For an undamaged element, there were seven degrees of freedom (dof) at one node (i.e., $u_{0},v_{0},w_{0},w_{0,x},w_{0,y},\psi_{x},\psi_{y}$) and 12 dof at one node of the delaminated/debonded element (i.e., $u_{0},v_{0},w_{0},w_{0,x},w_{0,y},\psi_{x},\psi_{y},{\bar{u}}_{x}^{j},{\bar{u}}_{y}^{j},{\bar{w}}^{j},{\bar{w}}_{,x}^{j},{\bar{w}}_{,y}^{j}$). The additional dof at the damaged nodes accounted for possible in-plane slippage and out-of-plane jumps at the delaminated/debonded interfaces.

For the active vibration control of the smart composite laminate, we employed a constant gain velocity feedback control (CGVF) strategy. We used a reduced-order state-space model to assess the vibration-suppression characteristics of the CGVF controller in the presence of sensor partial debonding and structural delamination. Equation (5) shows the reduced-order form of the governing equations of motion:

(5a) $$\bar{M}\ddot{r}+\bar{C}\dot{r}+\bar{K}r={\bar{F}}_{u}-{\bar{F}}_{\varphi }$$

(5b) $$\bar{M}=\emptyset^{T}M\emptyset =I$$

(5c) $$\bar{C}=\emptyset^{T}C_{u}\emptyset =diag\left[\begin{array}{ccc} 2\xi_{1}\omega_{1} & \cdots & 2\xi_{m}\omega_{m} \end{array}\right]$$

(5d) $$\bar{K}=\emptyset^{T}K\emptyset =diag\left[\begin{array}{ccc} \omega_{1}^{2} & \cdots & \omega_{m}^{2} \end{array}\right]$$

(5e) $${\bar{F}}_{u}=\emptyset^{T}F_{u}$$

(5f) $${\bar{F}}_{\varphi }=\emptyset^{T}K_{u\varphi }K_{\varphi \varphi }^{-1}F_{\varphi }$$

where r and ∅ refers to the modal coordinate vector and matrix of first *m* eigenvectors, respectively. The terms ξ and ω denotes the modal damping and natural frequencies for the first *m* modes. 

The reduced-order model-based state-space form that was employed for the modal control is shown by Equations (6):

(6a) $$\dot{X}=A_{c}X+B_{u}u+B_{\varphi }u_{\varphi }$$

(6b) $$X=\left\{\begin{array}{c} r\\ \dot{r} \end{array}\right\},\;A_{c}=\left[\begin{array}{cc} 0 & I\\ -\bar{K} & -\bar{C} \end{array}\right],\;B_{u}=\left[\begin{array}{c} 0\\ \emptyset^{T} \end{array}\right],\;B_{\varphi }=\left[\begin{array}{c} 0\\ -\emptyset^{T}K_{u\varphi }K_{\varphi \varphi }^{-1} \end{array}\right]$$

where, $A_{c}$, $B_{u}$, $B_{\varphi }$ denote the system characteristics matrix, mechanical input influence matrix and electrical input influence matrix, respectively. The terms *u* and $u_{\varphi }$ denote the mechanical and electrical input vectors, respectively.

The CGVF controlled system is given by Equations (7):

(7a) $$\dot{X}=\bar{A}X+B_{u}u_{u}$$

(7b) $$\bar{A}=\left(A-B_{\varphi }GC_{\varphi 1}\right)$$

where, *G* and $C_{\varphi 1}$ are the velocity control gain and output velocity influence matrix, respectively.

## 3. Numerical Results and Discussion

**Figure 2:**
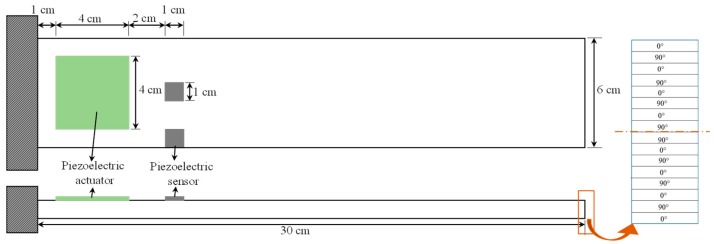
Geometric configuration of single-input-multi-output system for numerical simulation: (LHS) front and top view; (RHS) exaggerated view of the thickness direction.

In this study, a single-input-multi-output (SIMO) system is used to demonstrate the effects of sensor partial debonding and structural delamination on the active vibration control of a smart laminated composite plate. The system consists of a cross-ply laminated composite plate with two piezoelectric sensors and one piezoelectric actuator, as shown in Figure 2.

The cantilevered plate is made of 16 layers with symmetric cross-ply configuration, and the piezoelectric patches of PZT-5H are bonded to the surface of the plate. To account for both the bending and the twisting modes in the feedback control, one of the sensors is attached at the center of the plate, and the other is bonded at the edge side. The sensor at the off-center position was labelled sensor 1, and the sensor at the center of the plate was labelled sensor 2. Table 1 lists the material and geometric properties of a single lamina of the host laminated plate and PZT-5H.

**Figure 3:**
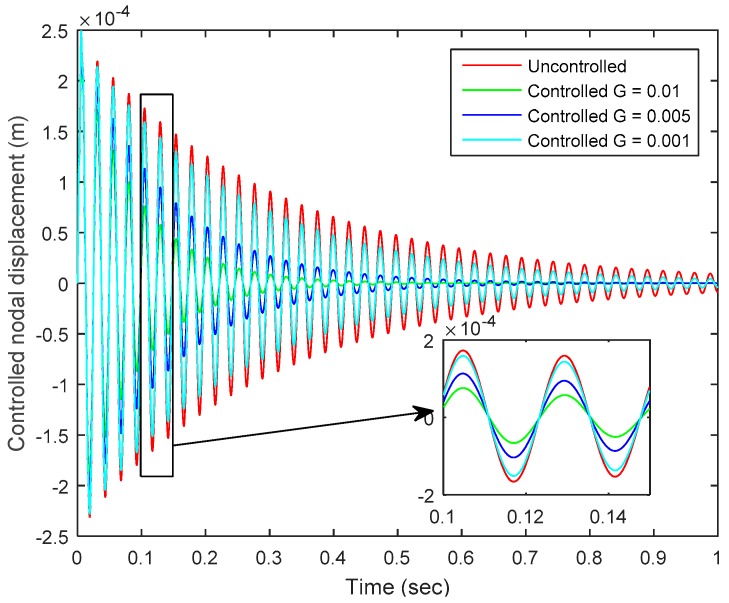
Controlled and uncontrolled nodal displacements at the tip of the smart plate.

The dynamic response of the smart plate was obtained by applying an impulse load of 1 N at the tip-center of the plate, and CGVF control strategy was implemented to suppress the vibration of the smart plate. Figure 3 shows the controlled and uncontrolled nodal displacements of the smart plate as measured at tip-center of the plate, which is well-controlled; increasing the value of the control gain (*G*) increases the control authority.

### 3.1. Effect of Partial Debonding of Sensor

**Figure 4:**
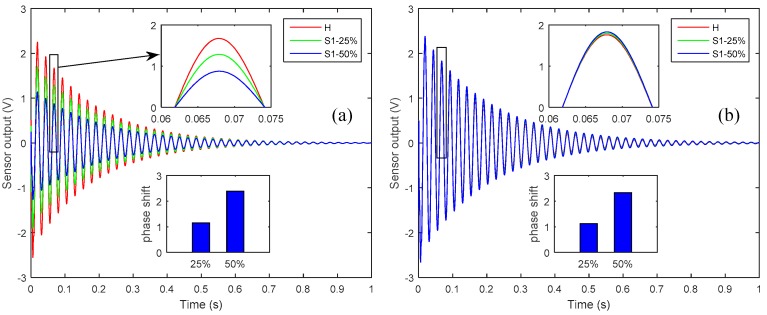
The effect of partial debonding failure of sensor 1 on the controlled response: (**a**) sensor 1 output, when sensor 1 is partially debonded; (**b**) sensor 2 output, when sensor 1 is partially debonded.

To investigate the effects of sensor debonding failure, a partial debonding of 25% and 50% were presumed at the right-side edge of sensor 1. The smart plate without any defects was termed as ‘healthy’ case and was used as a reference. Figure 4 shows the effect of partial debonding failure of the sensor 1 on the controlled response of the smart plate as measured by sensor 1 and sensor 2, compared with the reference case.

From Figure 4 one can see that partial debonding in a sensor diminishes the voltage measured by that particular sensor and increases the voltage output of the sensor that is perfectly bonded to the host structure. The decrease in the voltage output of the debonded sensor could be explained in terms of the working physics of the piezoelectric patch as a sensor. A piezoelectric patch generates a voltage signal when deformed mechanically, and the amplitude of the generated voltage is proportional to the mechanically induced strain. The presence of partial debonding in the sensor diminishes the mechanical strain induced in the sensor patch and so diminishes the voltage output of the debonded sensor.

**Figure 5:**
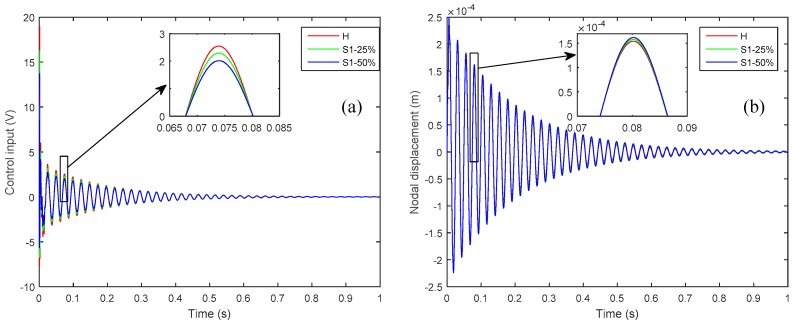
The effect of 25% and 50% partial debonding of sensor 1 on: (**a**) Control input signal; (**b**) Nodal displacement at the tip of the plate.

On the other hand, the voltage signal that is measured by the perfectly bonded sensor increases because of the diminishing of the control input signal applied through the actuator for vibration attenuation. The control input signal from the CGVF controller for attenuating the vibration is produced by the sensor’s output and control gain. If any sensor is partially debonded, the resulting control input signal is smaller in amplitude, which leads to a vibration that is larger in amplitude than the case when all the sensors are perfectly bonded; consequently the larger amplitude vibration results in a larger voltage output of the perfectly bonded sensor. The control input signal applied to the piezoelectric actuator and the controlled nodal displacement at the tip of the plate, as shown in Figure 5, confirms the diminishing of the control input signal and the augmentation of the vibration amplitude when there is a partial debonding of 25% and 50% between the host laminate and sensor 1.

From the subplots in Figure 4, one can see that the phase shift relative to the healthy case could be used as a damage severity index for the partially debonded sensor.

### 3.2. Effect of Structural Delaminations

**Figure 6:**
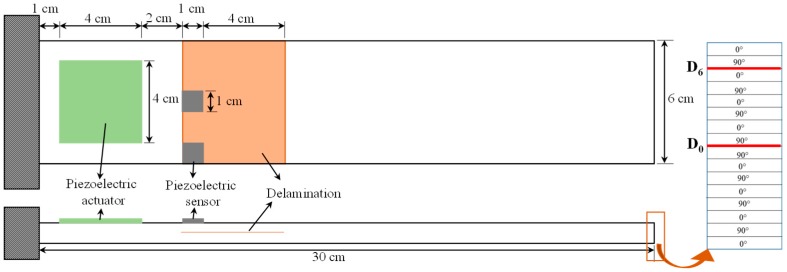
Geometry of (**left**): Smart composite laminate with seeded delamination; (**right**): optional interfaces of delamination through the thickness direction.

To study the effects of structural delamination on the active vibration control of a smart plate, a structural delamination of 5 cm is seeded at the mid-plane interface (*D*_0_) and at the 6th interface from the mid-plane (*D*_6_) along the thickness of the smart plate, as shown in Figure 6.

**Figure 7:**
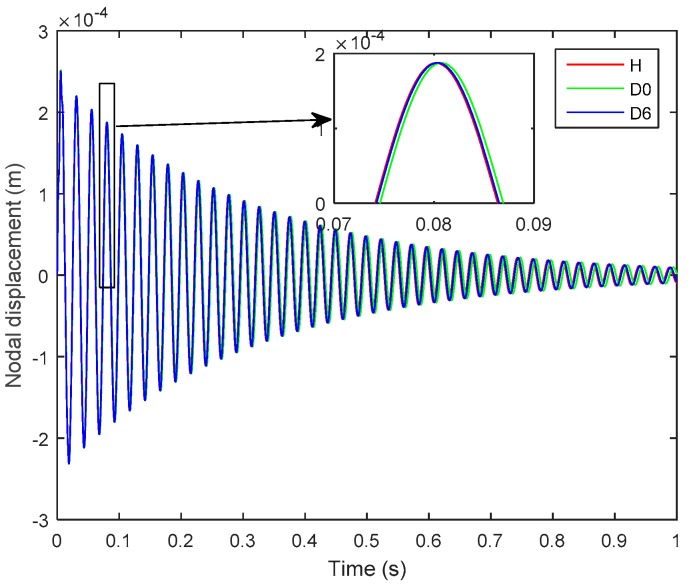
Open-loop nodal displacement at the tip of the plate for the healthy and delaminated smart plates (*D*_0_, *D*_6_).

**Figure 8:**
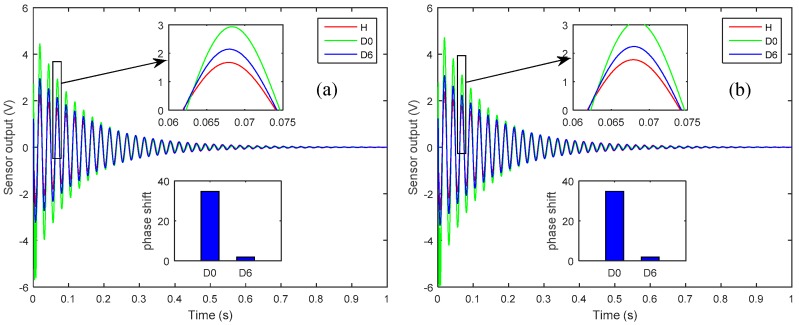
Closed-loop responses of the smart plates for the healthy and delaminated cases (*D*_0_, *D*_6_) as measured by: (**a**) Sensor 1; (**b**) Sensor 2.

It is worth noting that the presence of delamination at the mid-plane interface of the laminated plate is more severe and is more easily detectable than is a delamination of the same size that occurs farther away from the mid-plane, i.e., near the free surface [11]. Hence, in Figure 6, the *D*_0_ is more pronounced and more easily detected than is the *D*_6_. An impulse load of 1 N was applied at the tip of the plate for 0.01 s, and Figure 7 and Figure 8, respectively show the open-loop nodal displacement at the tip of the plate and the closed-loop response signals of the two sensors for the healthy and delaminated plates.

Herein, one can see from Figure 7 and Figure 8 that, although the presence of structural delamination has not altered the magnitude of the open-loop nodal displacement, the closed-loop sensor’s outputs clearly capture the effect of delamination in the laminated plate. Also, the closed-loop response for the healthy case is smaller in amplitude than is that for the delaminated cases, because the presence of structural delamination causes a loss of structural stiffness in the laminated plate [42]. Furthermore, the effect of more severe delamination (*D*_0_) is more pronounced than is that from the relatively less severe case of delamination (*D*_6_). The subplots in Figure 8 show that a phase shift relative to the undelaminated smart plate could be used to assess the level of severity of the delamination damage in the structure. 

**Figure 9:**
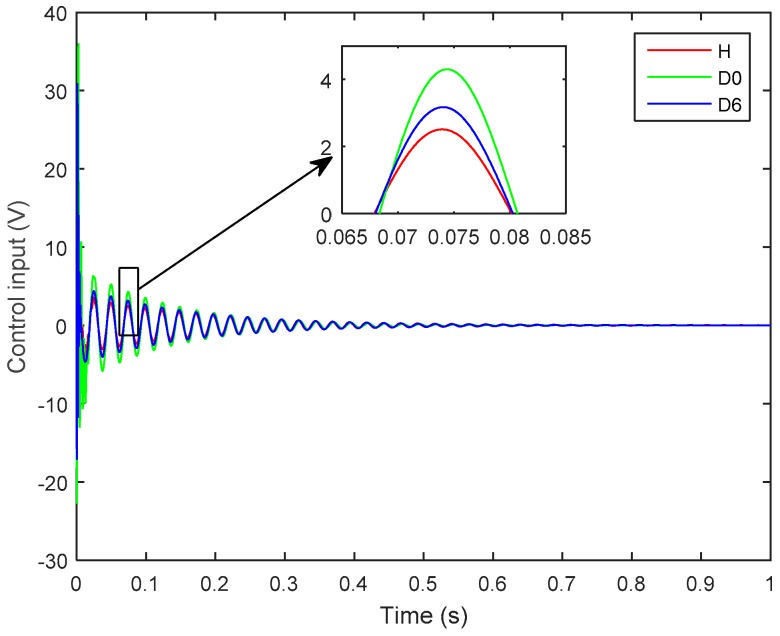
Control input signals as applied to control the healthy (H) and delaminated structures (*D*_0_, *D*_6_).

Figure 9 shows the effect of structural delamination on the control input signal applied to the actuator to attenuate vibration. Herein, one can see that the magnitude of the control input signal is the smallest for the healthy case (H), whereas higher control input voltages are applied to control the delaminated structures. Furthermore, the more severe case of delaminations (*D*_0_) requires a higher-magnitude control input than does the less severe case of delamination (*D*_6_).

**Figure 10:**
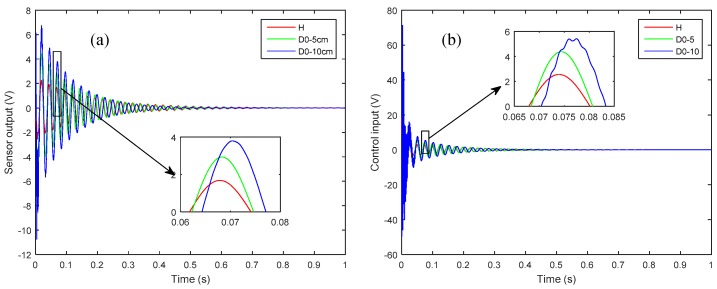
The effect of delamination length on: (**a**) closed-loop response as measured by sensor 1; (**b**) control input signal.

To study the effect of size of delamination on the active vibration control, the 5 cm delaminated case (*D*_0_-5) of Figure 6 is compared with a 10 cm delamination (*D*_0_-10) at the same interface and the same starting position at the left side of the plate. Figure 10 shows the effect of the two lengths of delamination on the controlled response as measured by sensor 1 and the control input signal.

Herein, it is observed that larger size delamination has more pronounced effects in terms of loss of structural stiffness and control input. Also, From Figure 10, the effect of phase shift can be easily observed for the larger size delamination.

### 3.3. Comparison of Sensor Partial Debonding and Structural Delamination

**Figure 11:**
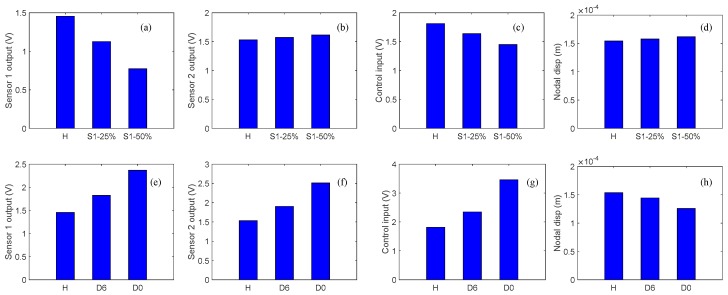
Comparison of the results for sensor debonding and structural delamination at a particular instant of time: (**a**) Sensor 1 output at *t* = 0.0925 s, sensor 1 debonding; (**b**) Sensor 2 output at *t* = 0.0925 s, sensor 1 debonding; (**c**) Control input at *t* = 0.072 s, sensor 1 debonding; (**d**) Controlled nodal displacement at *t* = 0.0805 s, sensor 1 debonding; (**e**) Sensor 1 output at *t* = 0.0925 s, structural delamination; (**f**) Sensor 2 output at *t* = 0.0925 s, structural delamination; (**g**) Control input at *t* = 0.072 s, structural delamination; (**h**) Controlled nodal displacement at *t* = 0.0805 s, structural delamination.

In order to investigate the effects of sensor partial debonding and structural delamination on the active vibration control of a smart composite laminate, we compared the controlled outputs of the sensors, control input signals, and controlled nodal displacements for the two types of damage at a particular instant of time, as shown in Figure 11a–h.

**Figure 12:**
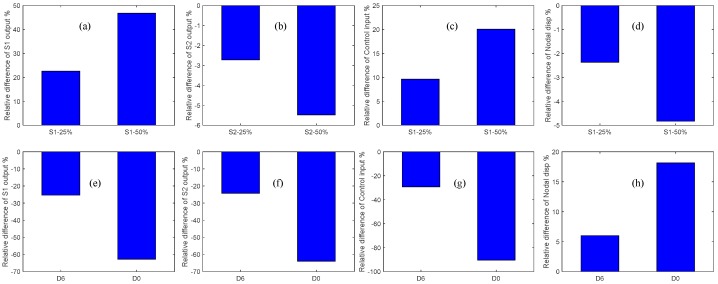
Comparison of the relative differences of sensor debonding and structural delamination as compared with the healthy state: (**a**) Sensor 1 output at *t* = 0.0925 s, sensor 1 debonding; (**b**) Sensor 2 output at *t* = 0.0925 s, sensor 1 debonding; (**c**) Control input at *t* = 0.072 s, sensor 1 debonding; (**d**) Controlled nodal displacement at *t* = 0.0805 s, sensor 1 debonding; (**e**) Sensor 1 output at *t* = 0.0925 s, structural delamination; (**f**) Sensor 2 output at *t* = 0.0925 s, structural delamination; (**g**) Control input at *t* = 0.072 s, structural delamination; (**h**) Controlled nodal displacement at *t* = 0.0805 s, structural delamination.

Herein, from Figure 11a,e one can see that the closed-loop response signal of the partially debonded sensor and delaminated structure follows a trend opposite to that of the healthy case. For instance, the closed-loop response of the partially debonded sensor decreases with the increasing length of the partial sensor debonding, whereas the closed-loop response increases with the increasing level of structural delamination. From Figure 11b,f, an identical trend can be seen in the controlled response signals as measured by the perfectly bonded sensor for the two types of damage (sensor debonding and structural delamination); however, the control input signal recovers the opposite trend for the two types of damage (Figure 11c,g). By comparing Figure 11d,h, one can see that sensor partial debonding and structural delamination have opposite effects in terms of the ability of the CGVF controller to suppress vibration. The same trend is observed from the relative differences of the debonded/delaminated cases as compared with the healthy case; as shown in Figure 12a–h.

From the effects of sensor partial sensor debonding and structural delamination on the control input and closed-loop nodal displacements (Figure 11c,d,g,h and Figure 12c,d,g,h), it is obvious that the presence of partial debonding in the sensor diminishes the ability of the CGVF controller to suppress vibration and may lead to an unstable controlled system. However, the presence of a structural delamination in the host laminate increases the control authority of the controller because of the loss of structural stiffness with delamination. The study could be extended to the active vibration control of the wings and vertical stabilizer of the aircraft [43,44].

For the experimental verification of the current work, the presence of system and surrounding noise, the preparation of composite coupons with the exact size of delamination, and emulating partial debonding in the small size sensor would pose potential difficulties and uncertainties. Also, in the current work, the relative effects of the single discrete delamination in the host structure and partial debonding failure of the sensor on the active vibration control of a smart composite laminate are studied separately. Investigating active vibration control in the presence of multiple discrete delaminations and their co-occurrence with sensor partial debonding would be a potential future research topic.

## 4. Conclusions

In this work, the effects of sensor partial debonding and structural delamination on the active vibration control of a piezo-bonded laminated composite are evaluated in a numerical framework. The damage models for a partially debonded sensor and structural delamination are developed in terms of a layerwise displacement field, a higher-order electric potential field, and generalized finite-element method. The electromechanically coupled governing equation of the problem was reduced in order by means of a generalized modal-order reduction method and was transformed into a state-space form. A constant gain velocity feedback controller is incorporated to suppress vibrations of the smart structure in the pristine and damaged states. The numerical results may be summarized by the following points:

(1) Sensor partial debonding and structural delamination show opposite trends in terms of the control input signals being applied to the actuator and the controlled nodal displacements.
(2) The presence of partial debonding between the piezoelectric sensor and host laminate causes the control input signals to reduce in magnitude and the controlled nodal displacements to increase in magnitude, whereas the presence of delamination damage in the host laminate increases the control input signals and diminishes the controlled nodal displacements.
(3) From a control standpoint, a partially debonded sensor may lead to an unstable controlled system because of the loss of control authority, whereas a delaminated structure may enable the controller to better suppress the vibrations. Also, it is worth noting that the presence of a large structural delamination may cause the smart structure to vibrate with an unboundedly larger amplitude vibration because of the larger amplitude of the control input signal.
(4) Relative phase difference could be used as an index for assessing the severity of sensor partial debonding and structural delamination.

## Figures and Tables

**Table 1:** Material and geometric properties of a single lamina of the host laminate and PZT-5H.

Properties	Host Lamina	PZT-5H
Young’s modulus (GPa)	*E*_1_=372, *E*_2_ = *E*_3_ = 4.12	62
Shear modulus (GPa)	*G*_12_ = *G*_13_ = 3.99, *G*_23_ = 3.6	*G* = 23.67
Poisson’s ration	*ν*_12_ = *ν*_13_ = 0.275, *ν*_23_ = 0.42	*ν* = 0.31
Density (kg/m^3^)	1788.5	7500
Piezoelectric constant (m/V)	-	*d*_31_ = *d*_32_ = −274 × 10^−12^
-	-	*d*_24_ = *d*_15_ = 741 × 10^−12^
Permittivity (nF/m)	-	*b*_11_ = *b*_22_ = *b*_33_ = 2.638
Length (m)	0.3	0.01
Width (m)	0.06	0.01
Thickness (m)	0.125×10^−3^	0.25 × 10^−3^
